# Clinical, laboratory and ultrasonographic findings at baseline predict long-term outcome of polymyalgia rheumatica: a multicentric retrospective study: Polymyalgia rheumatica predicted by ultrasonographic findings polymyalgia rheumatica outcome predicted early by ultrasound

**DOI:** 10.1007/s11739-023-03373-x

**Published:** 2023-07-27

**Authors:** Edoardo Conticini, Paolo Falsetti, Miriana d’Alessandro, Suhel Gabriele Al Khayyat, Silvia Grazzini, Caterina Baldi, Caterina Acciai, Stefano Gentileschi, Roberto D’Alessandro, Francesca Bellisai, Giovanni Biasi, Cristiana Barreca, Elena Bargagli, Luca Cantarini, Bruno Frediani

**Affiliations:** 1 Department of Medicine, Surgery and Neurosciences, Rheumatology Unit, University of Siena, Viale Mario Bracci, 53100 Siena, Italy; 2 Respiratory Diseases and Lung Transplantation Unit, Department of Medicine, Surgery and Neurosciences, University of Siena, Viale Mario Bracci, 16, 53100 Siena, Italy; 3 Neurorehabilitation Unit, San Donato Hospital, 52100 Arezzo, Italy

**Keywords:** Polymyalgia rheumatica, Giant cell arteritis, Ultrasonography, Arthritis

## Abstract

To assess the rate of PMR who, during the follow-up, undergo a diagnostic shift as well as to assess which clinical, laboratory and US findings are associated to a diagnostic shift and predict the long-term evolution of PMR. All PMR followed-up for at least 12 months were included. According to the US procedures performed at diagnosis, patients were subdivided into four subgroups. Clinical data from follow-up visits at 12, 24, 48 and 60 months, including a diagnostic shift, the number of relapses and immunosuppressive and steroid treatment, were recorded. A total of 201 patients were included. During the follow-up, up to 60% had a change in diagnosis. Bilateral LHBT was associated with persistence in PMR diagnosis, whereas GH synovitis and RF positivity to a diagnostic shift. Patients undergoing diagnostic shift had a higher frequency of GH synovitis, shoulder PD, higher CRP, WBC, PLT and Hb and longer time to achieve remission, while those maintaining diagnosis had bilateral exudative LHBT and SA-SD bursitis, higher ESR, lower Hb and shorter time to remission. Cluster analysis identified a subgroup of older patients, with lower CRP, WBC, PLT and Hb, lower PD signal or peripheral synovitis who had a higher persistence in PMR diagnosis, suffered from more flares and took more GCs. Most PMR have their diagnosis changed during follow-up. The early use of the US is associated with a lower dosage of GCs. Patients with a definite subset of clinical, laboratory and US findings seem to be more prone to maintain the diagnosis of PMR.

**Supplementary Information:**

The online version contains supplementary material available at 10.1007/s11739-023-03373-x.

## Introduction

Polymyalgia rheumatica (PMR) is a common condition characterized by the inflammatory involvement of shoulders, neck and pelvic girdle. Diagnosis is clinical, but ultrasonography (US) has recently gained more importance [1, 2] and is included in 2012 ACR/EULAR provisional classification criteria [3].

Therapy relies on glucocorticoids (GCs) which provide prompt relief and lead to markers of inflammation normalization. Nevertheless, despite being considered a benign, not life-threatening disease, PMR is burdened by significant morbidity, largely related to long-term GCs side effects [4], and relapses occur in up to 43–44% of patients [5, 6].

At the same time, PMR may be associated with different conditions, occurring at the time of diagnosis of PMR or representing its long-term complication. PMR may herald, even for years, the onset of giant cell arteritis (GCA) and up to 20% of patients with PMR may suffer from an asymptomatic, underlying, vasculitis [7]. A recent PET-CT study even reported 61% of subclinical GCA in refractory PMR [8].

On the other hand, PMR may represent the treacherous symptom of a seronegative elderly onset rheumatoid arthritis (EORA) [9, 10]. Moreover, evidence of calcium pyrophosphate dihydrate deposition disease (CPPD) can be found in up to 50% of PMR patients [11] and atypical late-onset spondylarthritis (EOSpA) could not be easily discriminated from PMR [12, 13].

Finally, PMR may evolve in a minimal disease activity condition, requiring low and persistent doses of GCs and flaring at every attempt to further reduce or withdraw the steroids [14]. In this subset of patients, usually elderly, adrenal failure may explain the persistent need for steroids [15–18].

Although a diagnostic shift is reported in up to two thirds of patients after long-term follow-up [6, 8, 10, 19–21], no definite clinical, laboratory or imaging finding has been identified to predict prognosis and different clinical course of PMR [22, 23], nor the risk of further relapses [5, 6, 24].

Therefore, in real life, PMR patients often remain in a long-term GC treatment, with several relapses, and experience a late change in its diagnosis only when referred to specialistic care settings [20, 22, 25].

In this regard, we aimed to retrospectively explore the landscape of PMR, in a large and heterogeneous cohort of patients with a long-term follow-up, also including the precocious use of diagnostic US, the onset of relapse and the optimal management in terms of treatment and diagnosis.

Primary endpoint of the study was therefore to assess the rate of PMR patients who, during the follow-up, undergo a different diagnosis (GCA, arthritis), as well as to assess which early clinical, laboratory and US findings are associated with a diagnostic shift and are able to predict the long-term evolution of PMR.

Secondary endpoints were to assess whether the precocious use of DMARD is associated with a lower dosage of GCs and a diagnostic shift, as well as to assess the optimal use of musculoskeletal and arterial US in the early diagnostic assessment of PMR.

## Methods

All patients were diagnosed with PMR in Rheumatology Unit, University of Siena, and other four clinics in south-eastern Tuscany and Umbria, Italy, from January 2017 to January 2022, were included.

Inclusion criteria were the fulfilment of Bird criteria [26], the availability of a minimum core set measures and follow-up data of at least 12 months from the first diagnosis.

Exclusion criteria were a previous diagnosis of PMR and of any other inflammatory rheumatic disease, the presence of symptoms of cranial GCA (amaurosis, headache, jaw or tongue claudication), a diagnosis of adrenal failure, concomitant treatment with oral GCs or any immunosuppressant and the lack of the minimum core set measures, as well as an inadequate follow-up duration.

### Clinical data

At baseline the following data were recorded: age, sex, fulfilling of ACR/EULAR criteria [3], with and without US, in addition to Bird criteria [26], with the presence of any of the following sign or symptoms: shoulder pain, shoulder tenderness, onset from less than 2 weeks, morning stiffness > 1 h, age > 65 years, depression or loss of weight, fever, as well as the presence of any trigger (i.e. infection, vaccine, oncologic disease or other).

### Laboratory data

At baseline the following findings were recorded: ESR, C-reactive protein (CRP), anti-nuclear antibodies (ANA), rheumatoid factor (RF) and anti-citrullinated peptides antibodies (ACPA) positivity, white blood cells, platelets, haemoglobin (Hb) and urate.

### Ultrasound examination

Only if performed at the time of the first diagnosis, musculoskeletal and axillary (AxA) and temporal arteries (TA) findings, according to EULAR/OMERACT guidelines [27, 28] were recorded.

US findings were recorded as follows: gleno-humeral (GH) effusion (JE)/synovial hypertrophy (SH), subacromion-subdeltoid (SA-SD) bursitis, long head of biceps tendon tenosynovitis (LHBT), wrist JE/SH, power Doppler (PD) signal on shoulder (any site) and wrist, metacarpophalangeal JE/SH [27], hip JE/SH, trochanteric bursitis, knee JE/SH, calcifications suggestive for CPPD or gout, evidence of enthesitis [28]. Joint erosions were recorded (for shoulder, wrist, MCP) only if undoubtfully related to local synovitis. Given the retrospective nature of the study, the US data were collected with a simplified method; grey-scale US data were collected as a whole for JE and SH, grading the articular involvement in a three-point scale (0 = absence of abnormalities, 1 = monolateral JE or SH, 2 = bilateral JE or SH), whereas information on PD signal was collected only for shoulder and wrist, with a three-point scale (0 = absence of PD signal, 1 = monolateral PD signals, 2 = bilateral PD signals).

Intima-media thickness (IMT) of both axillary (AxA) and temporal arteries (TA) (common, parietal, and frontal branches), as well as the low compressibility of TA and the presence of “halo sign” were recorded, too. AxA and TA US findings were recorded dichotomously, as negative or positive for GCA, according to the cut-off values proposed by Schmidt [29].

In Siena University Hospital, US was performed using an Esaote (Genoa, Italy) MyLab Twice machine equipped with linear 4–13 and 6–18 MHz and convex 1–8 MHz transducers and an Esaote (Genoa, Italy) MyLab X8 eXP machine equipped with linear 4–15 and 8–24 MHz and convex 1–8 MHz transducers. In three territorial outpatient clinics PDUS was performed using an Esaote (Genova, Italy) MyLab 25 portable machine equipped with linear 6–18 MHz and convex 1–8 MHz transducers.

Standardised B-mode and Doppler settings were optimized for all examinations (factory preset of the machines for musculoskeletal or small parts). Doppler parameters were pulse repetition frequency within 500–750 Hz for musculoskeletal scans and 1500–2200 for TA and AxA, Doppler frequency adapted to deepness (generally within 7–11.1 MHz) and a color gain just under the artifacts limit.

All four sonographers were rheumatologists trained in the same university hospital, with several years of experience (between 5 and 20 years) in musculoskeletal US (MSUS) and color Doppler US (CDUS).

### PET

If performed, PET findings were reported as negative, suggestive for GCA and suggestive for PMR.

### Treatment

All patients were treated with oral GCs; steroid regimen was not standardized but conducted in accordance with the currently available recommendations [30, 32]. The early administration of a DMARD was recorded.

### Clinical assessment

Patients who, despite the fulfilment of Bird and/or ACR/EULAR criteria [3], received a diagnosis of GCA or arthritis other than PMR, as well as the one being treated at baseline with bDMARDs, were excluded from follow-up.

### Follow-up

The following data from follow-up visits at 12, 24, 48 and 60 months, when available, were recorded: current dosage of GCs (0 mg, ≤ 7.5 mg or > 7.5 mg of PDN or equivalent), immunosuppressive treatment (csDMARDs and/or bDMARDs), the number of flares (defined as an increase in either ESR or CRP, plus a flare of PMR clinical features with a response to GCs) during the previous 12 months and the definite diagnosis (PMR, GCA, EORA, EOSPA, CPPD). Patients who, during one of the follow-up visits, switched from PMR from any of the abovementioned diagnoses were excluded from further evaluations.

### Ethics

The study was conducted in accordance with the tenets of the Declaration of Helsinki, and the use of clinical data for research purposes was approved by the local Ethics Committee of the University of Siena (Reference No. 22271, “RHELABUS”).

### Statistical analysis

Results were expressed as mean ± standard deviation. For categorical variables, Fisher’s exact or Chi-squared tests were used to compare proportions between groups. Student’s t-test was used to compare the means of continuous variables between two groups when the distribution of data was normal and with Welch’s correction otherwise.

A non-parametric Kruskal–Wallis test was used to compare the means of continuous variables (clinical, laboratory, and US characteristics at baseline) among groups (final outcomes), while a Dwass–Steel–Critchlow–Fligner (DSCF) test was used for pairwise comparisons.

Non-parametric Spearman rank test was applied to correlate variables.

Multivariable linear regression was performed with all significant variables (clinical, laboratory, and US characteristics at baseline) entered in a backward stepwise way to identify which factors independently correlated with the dependent variable (various relevant final outcomes), and this was checked for multicollinearity.

Binomial logistic regression and receiver operating characteristic (ROC) curve analysis were used to determine the predictive diagnostic value of each US/clinical parameter in diagnosing a true PMR, with clinical long-term diagnosis used as a gold standard.

All collected continuous variables (obtained at the onset of the disease) were included in a hierarchical clustering analysis attempting to identify clearly demarcated groups within the overall population. The clusters obtained were then compared with descriptive statistics.

The level of statistical significance was set at a *p*-level of 0.05. Statistical analyses were performed using Jamovi 1.8.4 and XLSTAT2021 statistical packages.

## Results

### Clinical, serological and US findings at baseline

A total of 201 patients were included. Clinical and laboratory data are summarized in Tables 1 and 2.

**Table 1:** Demographic and serological features at baseline

No	201
Age (years)	73.4 ± 7.63
M/F	89/112
ESR (mm/h)	56.97 ± 25.15
CRP (mg/dl)	4.451 ± 3.783
RF + (UI/ml)	32/201 (15.7 ± 79.86)
ANA +	8/201
ACPA +	4/201
Uric Acid	4.660 ± 2.076
WBC	7552 ± 4223
PLT	326.9 ± 117.8
HB	12.75 ± 1.233

Abbreviations: *ACPA* Anti–citrullinated protein antibody, *ANA* antinuclear antibodies, *CRP* C reactive protein, *DMARD* disease-modifying antirheumatic drugs, *ESR* erythron sedimentation rate, *F* female, *M* male, *RF* rheumatoid factor

**Table 2:** Clinical features at baseline

	No	Percentage
ACR/EULAR clinical	185/188	98.4%
ACR/EULAR US	122/155	78.70%
Bird diagnostic +	201/201	100%
Shoulder pain	193/199	96.98%
Shoulder tenderness	176/188	93.61%
Onset < 2 weeks	76/194	39.17%
ESR > 40	146/188	77.65%
Stiffness > 1 h	170/198	85.85%
> 65 years	191/198	96.46%
Depression	55/196	28.06%
Fever	39/196	19.89%
Trigger	42/175 (19 infections, 15 vaccination, 8 other, 0 malignancy)	24% (10.85% infections, 8.57% vaccination, 4.57% other)
Time to remission (weeks)	21.08 ± 16.90	n.a

Abbreviations: *ESR* erythron sedimentation rate, *ACR/EULAR* American College of Rheumatology/European League Against Rheumatism, *US* ultrasonography

According to the US performed at baseline, which was not conducted on the ground of clinical suspicion but only in accordance with the personal experience and capabilities and in a real-life context (availability of an US machine, ability in performing musculoskeletal US, expertise in vascular US), patients were subdivided in four subgroups: in 32 diagnoses was clinical, without the use of US (group A); 35 underwent shoulders, hips, hands/wrists and knees US (group B); 48 underwent shoulders, hips, and TA/AxA CDUS (group C), while 86 had a complete US evaluation comprising shoulders, hips, hands/wrists, knees, heels and TA/AxA CDUS (group D).

At baseline, 14/134 (10.4%) patients from groups C and D displayed US evidence of GCA and were therefore excluded from follow-up. US findings are summarized in Table 3.

**Table 3:** US findings at baseline

	No	Percentage
Joint erosions (any site)	16/131	10%
SA-SD bursitis	98/165 (70 bilateral)	59.39%
GH JE/SH	93/161 (68 bilateral)	57.76% (42.23% bilateral)
LHBT tenosynovitis	125/161 (90 bilateral)	77.63% (55.9%)
PD-shoulder (any site)	49/149 (9 bilateral)	32.88% (6.04%)
Wrist JE/SH	39/145 (25 bilateral)	26.98% (17.24%)
PD-wrist (radio-carpal)	39/150 (25 bilateral)	26% (16.66% bilateral)
MCP JE/SH	20/138 (12 bilateral)	14.49% (8.69% bilateral)
Hip bursitis (any site)	36/136 (22 bilateral)	26.47% (16.17%)
Knee JE/SH	27/136 (10 bilateral)	19.85% (7.35% bilateral)
Calcifications suggestive for CPPD	31/140	21.98%
Enthesitis (heel, knee)	10/140 (4 bilateral)	7.14% (2.85% bilateral)
IMT AxA > 1 mm	12/132	9.09%
Pathological TA	10/132	7.57%

Abbreviations: *AxA* axillary arteries, *GH* gleno-humeral, *IMT* intima-media thickness, *JE* joint effusion, *LHBT* long head biceps tendón, *MCF* metacarpophalangeal, *PD* Power Doppler, *SA-SD* subacromion-subdeltoideal, *SH* synovial hypertrophy, *TA* temporal arteries

### Treatments and flares during follow-up

After 12 months, 80.3% patients remained in GC treatment (however only 5.8% with dosage > 7.5 mg/die), while the ratio was reduced to 48.8% at 24 and to 40.9% at 36 months.

In the first year of follow-up, 33.7% PMR patients suffered from one disease flare, while 16.5% had more than one (up to 5) and 49.7% remained in remission. In the long-term follow-up, PMR patients experienced flares in 25.8% of cases within 24 months and in 40.9% within 36.

Correlations between clinical and serological findings and the number of flares are reported in Supplementary material 1.

### Diagnostic shift during follow-up: frequencies and predictors

**Fig. 1:**
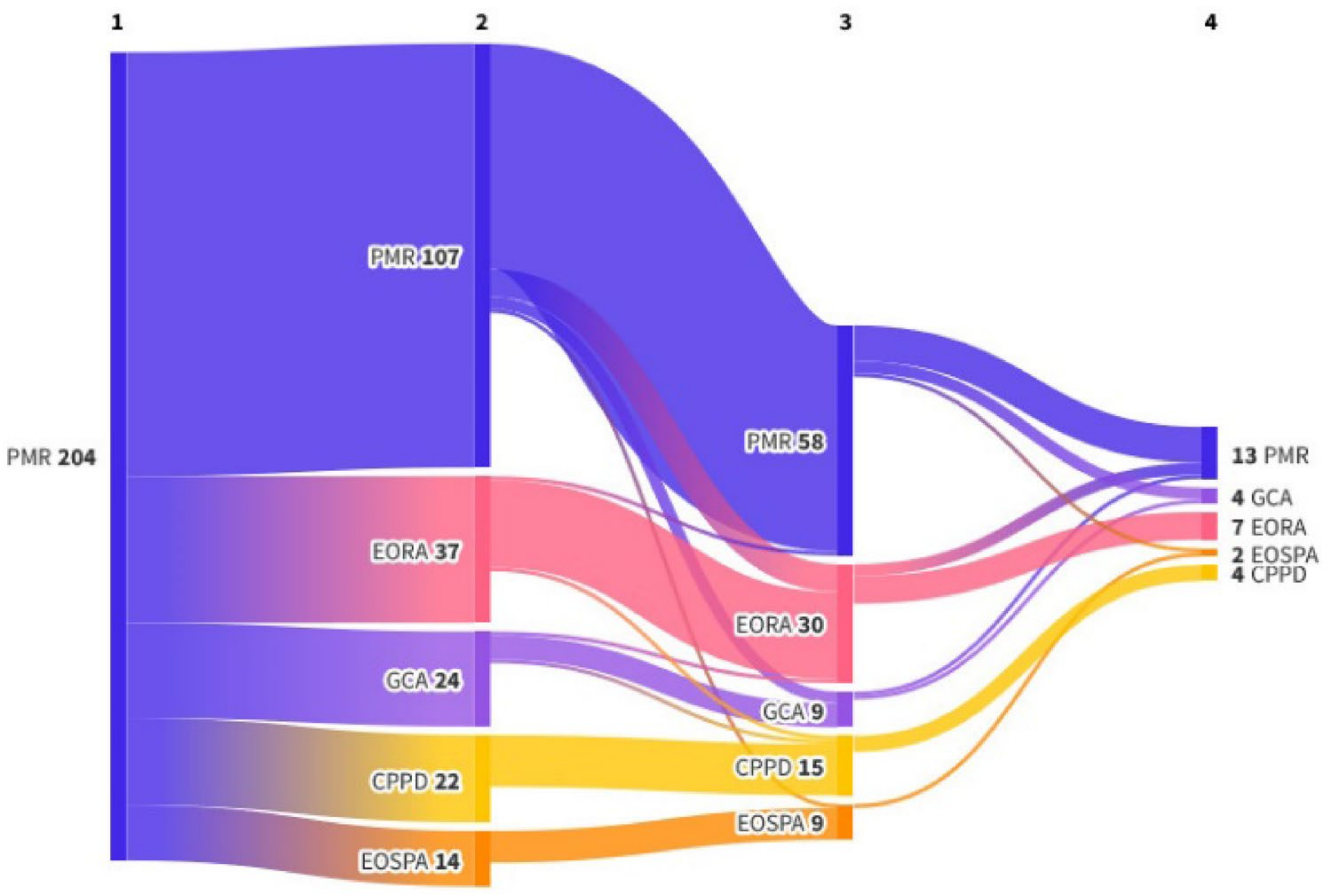
Diagnostic shift during follow-up. Abbreviations: *EORA* elderly onset rheumatoid arthritis, *EOSPA* elderly onset spondyloarthritis, *GCA* giant cell arteritis, *PMR* polymyalgia rheumatica

The follow-up study demonstrated that about half of the patients with a PMR-onset experienced a change in their diagnosis in the course of follow-up. In particular, within 12 months 47% had a diagnostic shift, within 24 months 52.8% had a change in diagnosis, and finally 60% of 19 patients followed for 36 months had a different diagnosis than PMR (Fig. 1).

The multivariate logistic regression showed that bilateral LHBT tenosynovitis at onset is the variable that better defines the persistence in PMR diagnosis (*p* = 0.05, OR 8.425), whereas gleno-humeral synovitis (*p* = 0.022, OR 0.074) and RF positivity (*p* = 0.028, OR 0.993) are the variables significantly associated to a diagnostic shift on the follow-up.

**Fig. 2:**
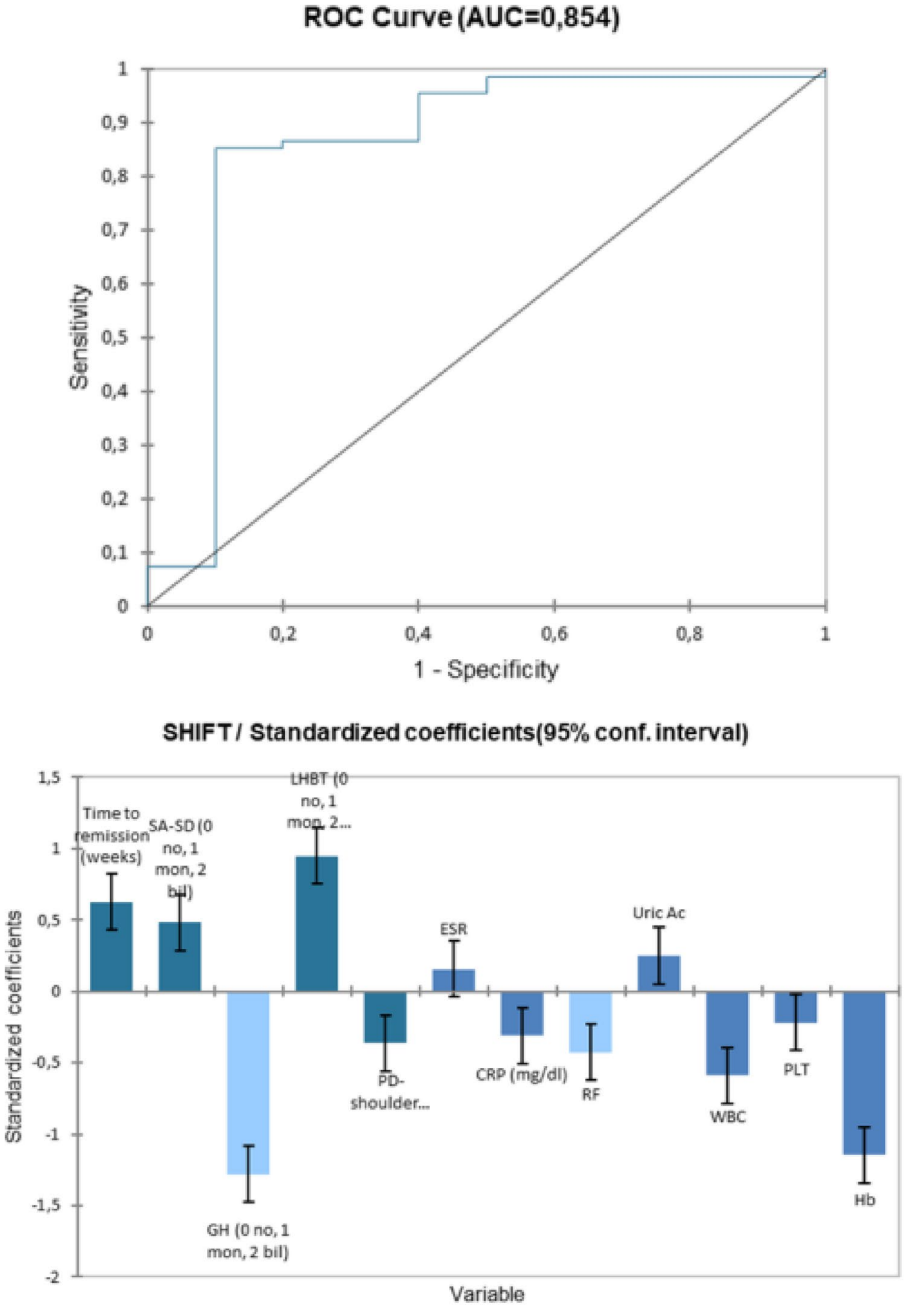
ROC curve and variables associate positively or negatively with diagnostic shift during follow-up

The model that better described (AUROC 0.854) (Fig. 2) a patient with a diagnostic shift comprised higher frequency of bilateral gleno-humeral synovitis, bilateral PD signals on shoulders (any site), higher values of CRP, WBC, PLT and haemoglobin, longer time to obtain remission.

On the other hand, the patients maintaining diagnosis of PMR had bilateral exudative LHBT tenosynovitis (OR 8.425) and SA-SD bursitis (OR 2.619), higher values of ESR (OR 1.015), lower values of haemoglobin (OR 0.428) and shorter time to remission (OR 1.076).

Finally, a diagnostic shift to GCA was positively predicted by fever and negatively by a rapid (< 2 weeks) onset of symptoms.

### Cluster analysis

All collected continuous variables were included in a hierarchical clustering analysis identifying two clearly demarcated groups (Fig. 1s), which were then phenotypically characterized in detail (Table 1s).

Cluster 2 identified older PMR patients, with lower systemic inflammation, lower levels of WBC, PLT and Hb, who had a higher persistence in PMR diagnosis at 12 (42.7% vs 29.3% of Cluster 1), 24 (37.2% vs 25.6% of Cluster 1) and 36 months (36.4% vs 21.2% of Cluster 1).

At baseline, these patients suffered more commonly from shoulder pain (61.3% vs 37.3%) and tenderness (57.5% vs 39.7%) and US displayed a lower PD signal (no PD signal on shoulders in 39.1% vs 27.5% of cluster 1, and no PD signal on wrists in 47.1% vs 29.4% of cluster 1) or less peripheral synovitis (no knee synovitis in 43.9% vs 33.3% of cluster 1, and no MCP synovitis in 50% vs 30.3% of cluster 1). An environmental trigger before onset was more commonly reported in cluster 2 (5,8% reported vaccinations before onset, vs 1.4% of cluster 1).

During follow-up, these patients suffered from more frequent flares at 12 (29.1% vs 15.3% of Cluster 1) and 24 months (12.3% vs 6.2% of Cluster 1) and were taking GC at 12 (51.4% vs 32% of Cluster 1) and 24 months (30.8% vs 9.2% of cluster 1).

### Impact of different diagnostic modalities on long-term clinical outcomes

The comparisons between groups B, C and D and group A showed significant differences in diagnosis at 12 (*p* = 0.0145) and 24 months (*p* = 0.0432), dosage of GC at 12 months (*p* = 0.0009) and bDMARDs at 12 (*p* = 0.0073) and 24 months (*p* = 0.0378).

DSCF pairwise comparisons test among the groups showed a higher dosage of GC at 12 months in group A, as demonstrated by the lower ratio of patients in GC-free treatment after one year from diagnosis in group A (6.4%, *p* = 0.002, Fisher test) when compared with groups D (12.9%), B (28.1%) and C (37.5%). Conversely, the ratio of patients in treatment with bDMARDs was significantly higher in group C (15%, *p* = 0.007, Fisher test).

No significant differences were evidenced in terms of DMARDs prescription nor number of disease flares, although a slightly not significant difference was assessed for flares at 24 months between groups A and D (*p* = 0.06). Indeed, 89 patients did not present any flare at 24 months: 83 of them underwent US at baseline.

The frequencies of diagnostic shifts varied among the different subgroups: among the patients diagnosed with GCA within the 12 months after diagnosis of PMR, 18 belonged to groups B, C and D, while only 2 to group A. Similarly, among the 18 diagnosed with CPPD, no one belonged to Group A, while 14 to Group D.

### Correlations between US findings and long-term clinical outcomes

A correlation analysis was performed between US findings at the onset and long-term outcomes.

At 12 months, persistence on GCs correlated only with absence of joint erosions (*p* = 0.013). Prescription of DMARDs correlated with the evidence of synovitis on wrists (*p* < 0.001) and MCP (*p* = 0.003), PD signals at wrists (*p* = 0.001), and presence of joint erosions (*p* < 0.001). Prescription of bDMARD strongly correlated (*p* < 0.001) with evidence of IMT > 1 AxA and TA halo. No US feature correlated with a number of flares at 12 months.

At 24 months, no US feature showed a correlation with persistence on GC. Prescription of DMARDs correlated with wrists (*p* < 0.001) and MCP (*p* = 0.005) synovitis, PD signals at wrists (*p* = 0.001), calcifications suggestive of CPPD (*p* < 0.009), and presence of enthesitis (*p* = 0.02). Prescription of bDMARD correlated with evidence of IMT > 1 AxA (*p* < 0.0031) and PD signals on shoulders (*p* = 0.002). No US feature correlated with the number of flares at 24 months.

US findings that better predicted the shift of PMR to EORA were GH synovitis (OR 0.074), SA-SD bursitis, wrist synovitis and wrist PD > 1. Conversely, no US findings, except for pathological AxA and/or TA ones, could predict the shift to GCA.

### Models of long-term clinical outcomes prediction (supplementary materials 2)

## Discussion

In our cohort, comprising 201 patients evaluated at baseline with 4 different management procedures, we found a relevant ratio of diagnostic shift, higher than 50%, during follow-up. This confirms similar findings reported in previous papers, which had assessed a change on diagnosis in up to 66% of PMR patients after long-term follow-up [6, 8, 10, 19, 21].

Such an uncertainty in achieving a “definite diagnosis” at first assessment, despite the formal fulfilment of diagnostic [26] and/or classification criteria [3], is probably due to the lack of gold-standard diagnostic procedures, to exclude mimickers [19, 33, 34].

Moreover, most patients are diagnosed and managed only in primary care settings, while only the ones with atypical presentation and/or more severe and difficult-to-treat disease are referred to secondary or tertiary centres [22, 35, 36]. This potentially induces spectrum biases about the course of PMR in relation to imaging procedures, treatment and outcome [1, 8, 11, 12, 37, 38].

At the same time, the complete withdrawal of GCs treatment is far from being achieved in the majority of patients, in which prolonged steroid assumption is one of the leading causes of morbidity [4]: also in our cohort, at 12 months only 19.7% of subjects were GCs-free and such a ratio, although significantly increased, remained higher than suggested by guidelines [30–32] at 36 months, when 40.9% of patients were still taking steroids. These findings do not differ from the ones from other cohorts [22], also in terms of relapses [5, 6] and prescription of DMARDs [6]: in our study, 50.3%, 25.8% and 40.9% of patients suffered from disease flare at 12, 24 and 36 months, respectively.

When trying to assess which clinical and laboratory findings are associated with disease flares, a positive, strong, correlation was evidenced with longer time for achieving remission and GCs dosage; similarly, patients taking a high dosage of prednisone at 12 months were more prone to take GCs at further follow-up visits. Such findings confirm that a prompt and complete response to GCs is a predictor of a better course of disease, burdened by few or no flare. While a longer time to remission may be explained by a more aggressive disease at baseline (higher CRP and lower Hb), the persistent need for steroids at 12, 24 and even 36 months is presumably due to other mechanisms. In this regard, if the possibility of a misdiagnosis, such as EORA, cannot be excluded, a condition of immune-endocrine senescence should be considered: adrenal failure may occur in active, untreated patients [15–17] and after both short and long-term steroid therapy [16–18, 39]. In these patients, GC therapy could mostly constitute an endocrinological supplementation.

Further analyses were carried out to assess which features may predict the persistence of PMR diagnosis or its shift. Patients maintaining diagnosis of PMR displayed bilateral LHBT, SA-SD bursitis, higher ESR, lower Hb and shorter time to remission, while the ones shifting to arthritis had RF positivity and GH synovitis. This is not fully surprising because, aside from US findings, PMR patients are often anemic and display a brilliant response to GCs. Nevertheless, these findings have an important clinical relevance: the evidence of GH synovitis, although comprised among ACR/EULAR criteria [3] should address the clinician to evaluate peripheral joints, particularly in the case of RF positivity, as they may herald a concomitant arthritis rather than a “pure” PMR. Similarly, the occurrence of fever and a subacute (> 2 weeks) onset of symptoms, both predicting GCA, should make the clinician carefully evaluate a subclinical GCA. The occurrence of subclinical large vessel vasculitis, far from being considered an unusual or only a late complication of PMR, is evidenced in up to 20% of patients [7, 40] and a recent meta-analysis, which is in line with our findings [40] has found that fever, and not CRP and ESR, is strongly associated (OR 1.83, 0.90–3.71) with GCA in PMR.

In line with the abovementioned evidence, clustering analysis supports the hypothesis that a peculiar subset of older subjects, with a lesser extent of GH and peripheral synovitis and lower inflammatory markers, could have a higher persistence on PMR diagnosis and suffer from a prolonged course, with more frequent flares, and dependence on low GC doses. A similar subset was described in a previous paper employing MRI, in which patients with less hip synovitis and more sever extracapsular hip involvement had a rapid response but a higher long-term persistence on GC [1]. Moreover, in line with a previous paper [41], cluster analysis confirmed a statistically significant more common occurrence of environmental trigger (usually vaccination) in this subset of patients, older, with less GH synovitis, short time to remission but with a persistent need for GCs.

The precocious use of US in patients with PMR has already demonstrated an increase of diagnostic specificity [3, 21, 42–44]. Our results show that the overall management of the disease does not change with the introduction of US at onset, as we could not demonstrate significant differences in the incidence of disease flares and prescription of DMARDs among the various management modalities (with or without US).

Nevertheless, the comparisons between groups B, C and D, employing US, and group A, in which diagnosis was only clinical, displayed showed significant differences. In particular, patients managed without US showed higher dosages of GC at 12 months and longer persistence on GC; this has paramount importance, as relevant morbidity of PMR is related to long-term GCs side effects [4].

Moreover, patients diagnosed without US were less prone to take bDMARDs at 12 and 24 months. This, as well as the low GCs dosage, is probably related to the precocious diagnosis of GCA, which was made only in those patients who underwent TA and AxA CDUS. Indeed, according to the protocol of treatment of our “Vasculitis clinic”, patients diagnosed with GCA are immediately treated with Tocilizumab and withdraw GCs after 6 months. This is corroborated by the evidence that the prescription of bDMARDs strongly correlated only with the presence of “halo sign” or pathological IMT at AxA and/or TA.

At the same time, CPPD, as well as GCA, was not diagnosed at baseline in any of the patients belonging to group A, displaying the role of US in enhancing diagnostic accuracy at baseline.

In addition, in terms of follow-up and long-term outcome, US displays interesting insights, although only a few papers had previously investigated the role of imaging procedures in PMR, displaying conflicting evidence. Some studies [45] evidenced a correlation between shoulder PD and a number of relapses, while others focused on soft tissue hypervascularization [43] or extrasynovial involvement [42] to increase the diagnostic specificity of ACR/EULAR criteria [3].

Differently from the abovementioned papers [45], we did not observe any correlation between US findings and the number of disease flares. Conversely, the persistence of GCs at 12 months strongly correlated with the absence of joint erosions, while the prescription of DMARDs at 12 and 24 months was predicted by erosions, wrists and hands synovitis and PD signal. That means that this subset of patients, displaying proliferative proximal and peripheral synovitis with PD signal is de facto composed of subjects with polymyalgic onset of an elderly onset arthritis, therefore needing a precocious administration of DMARDs.

In addiiton, when the statistical analysis was applied to assess long-term outcomes aside from diagnostic shift, the best regression model for disease flares and GC persistence comprised longer time to remission, lower serum Hb and no peripheral involvement, all features belonging to cluster 2, which better described patients less prone to a diagnostic shift.

The main limit of the study is the retrospective nature, as well as that the final diagnosis entrusts on a single judgement of a rheumatologist.

Another limit of the study is the unavailability of disease activity scores focused on pain, stiffness, fatigue and disability, as well as a detailed clinical examination of peripheral joints due to the retrospective design of the study. Third, we did not assess adrenal function: it is possible that some of those who were not able to wean off GCs actually suffered from adrenal failure. Fourth, the exclusion of patients lost at follow-up may have led to an under or overestimation of flares and diagnostic shift ratio. Fifth, although conceived as a real-life study, ours included also patients evaluated in a tertiary centre by rheumatologists highly skilled in US. The feasibility of an extensive US assessment is puzzling in daily clinical practice and further efforts should be addressed to define faster US protocols, targeted according to clinical and laboratory features. Finally, despite an overall adherence to the currently available guidelines, our patients, except for the ones affected by GCA who were referred to a single rheumatologist experienced in vasculitis, were not treated in the same manner: indeed, the withdraw of GCs and the prescription of DMARDs did not follow a standardized protocol and lied into clinicians’ preference and experience, including the capability of performing US.

In conclusion, our study strengthens the concept that PMR is a clinically heterogeneous disorder and should be potentially considered as a syndrome.

Despite the protean presentation and outcome, certain clinical, serological and imaging findings could precociously suggest a response to treatment, diagnostic shift and GCs dosage. In this regard, US has a potential value for being considered the missing tool for a more precise diagnosis of PMR.

Further efforts, and in particular prospective studies with homogeneous diagnostic and treatments schemes, should be therefore made to describe the multiple and heterogeneous phenotypes of polymyalgic syndrome at its earliest presentation, to set up the more appropriate treatment and management.

### Supplementary Information

Below is the link to the electronic supplementary material.

Supplementary file1 (DOCX 102 KB)

## Data Availability

The data presented in this study are available on request from the corresponding author.
