# Supplementary material for: Clinical, laboratory and ultrasonographic findings at baseline predict long-term outcome of polymyalgia rheumatica: a multicentric retrospective study: Polymyalgia rheumatica predicted by ultrasonographic findings polymyalgia rheumatica outcome predicted early by ultrasound
Source: Intern Emerg Med. 2023 Jul 27;18(7):1929–39. doi: 10.1007/s11739-023-03373-x (PMC10543828; doi:10.1007/s11739-023-03373-x)
Supplement: Supplementary file 1 — Supplementary file1 (DOCX 102 KB) [file 11739_2023_3373_MOESM1_ESM.docx]

**Supplementary materials 1**

**Treatments and flares during follow-up:**

After 12 months, 80.3% patients remained in GC treatment (however only 5.8% with dosage >7.5 mg/die), while the ratio was reduced to 48.8% at 24 and to 40.9% at 36 months. DMARDs, which were prescribed in 74/201 (36.8%) patients at baseline, were taken by 53.7% of them at 12 months, 40.5% at 24 months, and 81.8% at 36 months. bDMARDs, which were prescribed at baseline only in patients with a diagnosis of GCA, were taken by 5.9% of patients at 12 months; such a ratio remained stable (4.5%) at 36 months.

In the first year of follow-up, 33.7% PMR patients suffered from one disease flare, while 16.5% had more than one (up to 5) and 49.7% remained in remission. In the long-term follow-up, PMR patients experienced flares in 25.8% of cases within 24 months and in 40.9% within 36. The number of flares directly correlated to GC dosage (Spearman rho 0.302, *p*<0.001) and bDMARDs prescription (rho 0.169, *p*=0.021) at 12 months. Moreover, a positive, statistically significant, correlation was evidenced between GC dosage at 12 and 24 months (rho 0.284, *p*=0.002).

A direct correlation was evidenced between the number of flares at 12 and 24 months (*p*=0.032), as well as between flares themselves, longer time to remission (*p*<0.001) and higher dosage of GC at 12 and 24 months.

A longer time to remission strongly correlated also with higher CRP (*p* =0.013) and lower Hb (*p* =0.012) at baseline, while fever positively correlated with ESR (*p*<0.001) and systemic symptoms (*p* <0.001). No significant correlation was found for age at onset of symptoms.

**Supplementary materials 2**

**Models of long-term clinical outcomes prediction**: A backward stepwise multivariate regression analysis was applied to verify which clinical and US features at onset could predict long-term relevant outcomes (flares and persistence on GC therapy) independently from diagnostic shift.

The best regression model for prediction of flares at 12 months (r^2^=0.173, *p*=0.001) comprised longer time to remission (*p*=0.025), lower levels of haemoglobin (*p*=0.043) and low frequency of hip synovitis (*p*=0.024).

The best regression model for prediction of persistence on GC therapy at 12 months (r^2^=0.143, *p*=0.001) comprised longer time to remission (*p*=0.007), absence of joint erosions (*p*=0.005) and absence of wrist synovitis and PD signals (*p*=0.028).

The best regression model for prediction of persistence on GC therapy at 24 months (r^2^=0.212, *p*=0.004) comprised longer time to remission (*p*=0.05), presence of systemic symptoms (*p*=0.05), lower levels of haemoglobin and absence of fever (*p*=0.033), older age (*p*=0.009).

The best regression model for the prediction of a longer time to remission (r^2^=0.244, *p*<0.001) comprised absence of calcifications suggestive for CPDD (*p*=0.004), absence of LHBT tenosynovitis (*p*=0.018), lower levels of haemoglobin (*p*=0.003) and presence of SA-SD bursitis (*p*=0.03) and joint erosions (*p*=0.037).

**Figure 1s**. Cluster analysis


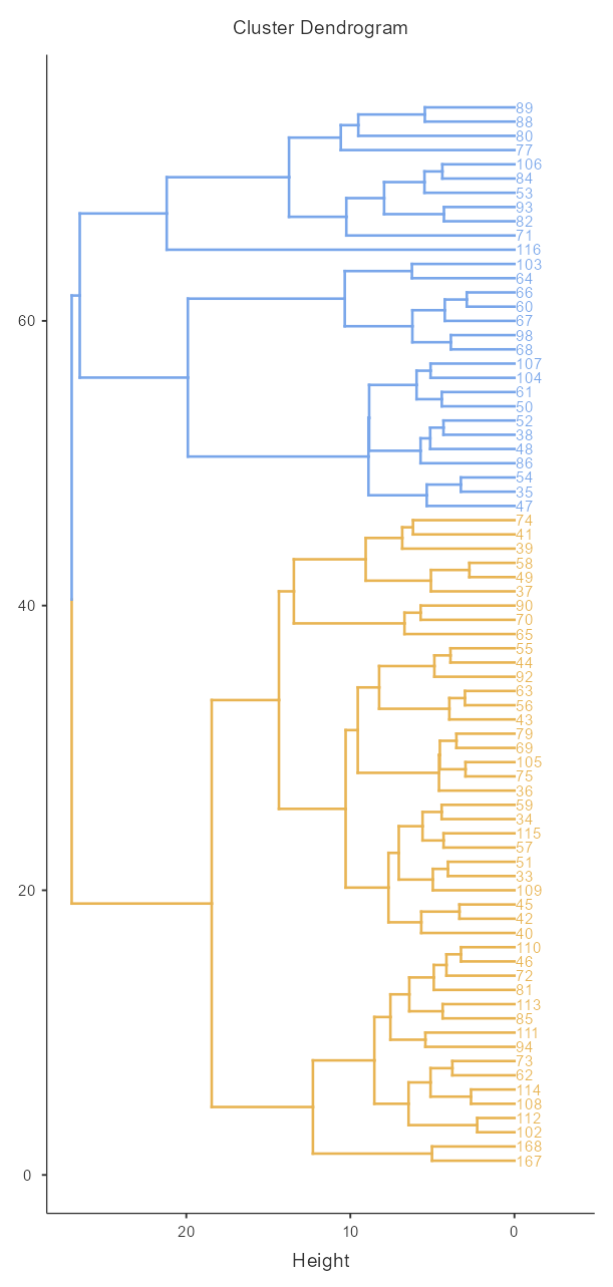


**Table 1s**: Descriptive characterization of the two groups from cluster analysis

|  | | | |
| --- | --- | --- | --- |
|  | A | Mean | SD |
| Age | cluster 1 | 71.31 | 6.67 |
|  | cluster 2 | 73.00 | 7.43 |
| ESR | cluster 1 | 59.86 | 27.00 |
|  | cluster 2 | 55.27 | 25.18 |
| CRP (mg/dl) | cluster 1 | 4.19 | 3.39 |
|  | cluster 2 | 4.05 | 3.84 |
| RF | cluster 1 | 14.45 | 30.14 |
|  | cluster 2 | 45.29 | 150.06 |
| Uric Ac | cluster 1 | 4.65 | 2.02 |
|  | cluster 2 | 4.51 | 1.98 |
| WBC | cluster 1 | 9457.76 | 2845.05 |
|  | cluster 2 | 7806.13 | 3894.72 |
| PLT | cluster 1 | 362.33 | 92.87 |
|  | cluster 2 | 304.68 | 145.92 |
| Hb | cluster 1 | 12.45 | 1.18 |
|  | cluster 2 | 11.66 | 3.57 |
|  | | | |

**List of abbreviations**: CRP: C reactive protein; ESR: erythron sedimentation rate; Hb: hemoglobin; PLT: platelets; RF: rheumatoid factor; WBC: white blood cells.
